# Neurological and neuropsychological correlates of Klippel-Feil syndrome

**DOI:** 10.1007/s10072-025-08454-7

**Published:** 2025-09-18

**Authors:** Sara Melchiorre, Mirella Russo, Matteo Santilli, Gaetano Polito, Consuelo Ciprietti, Dario Calisi, Valentina Panara, Loris Di Clemente, Astrid Thomas, Stefano L. Sensi

**Affiliations:** 1 Department of Neuroscience, Imaging, and Clinical Science, “G. D’Annunzio” University of Chieti-Pescara, Chieti, Italy; 2 Institute of Neurology, “SS Annunziata” Hospital -University of Chieti-Pescara, Chieti, Italy; 3 Molecular Neurology Unit, Center for Advanced Studies and Technology (CAST), University of Chieti-Pescara, Chieti, Italy; 4 Cognitive Neurology Unit, Institute for Advanced Biomedical Technologies (ITAB), University of Chieti-Pescara, Chieti, Italy

**Keywords:** Klippel-Feil syndrome, Retinotectal mapping, Vertebrae fusions, Neuroplasticity, Rare congenital malformation, Visuospatial impairment

## Abstract

**Background:**

Klippel-Feil syndrome is a rare congenital malformation caused by fusions of cervical vertebrae. In 50% of these patients, a triad of short neck, limited neck motion, and low posterior hairline characterizes the clinical presentation. In KFS, neurological deficits are common due to cervical canal stenosis and other deformities involving basicranial structures. Other congenital anomalies are also associated with the syndrome.

**Case presentation:**

Our case describes a particular case of KFS, showing a disconnection between a severe involvement of the cervical-occipital structures indicated by magnetic resonance imaging and a mild clinical presentation. Moreover, a slight visual-spatial deficit was found in neuropsychological tests. No prior association between KFS and visuospatial impairment has been reported.

**Discussion and conclusions:**

GDF6, a gene associated with KFS, plays a role in retinotectal mapping, which organizes visual stimuli in the brain. Early neurodevelopment abnormalities, such as atlanto-occipital anomalies in KFS, might affect related brain structures, which could explain the patient’s impaired visuospatial function. In addition, compensatory neuroplasticity underscores how the brain may adapt to congenital defects, even severe ones.

**Supplementary Information:**

The online version contains supplementary material available at 10.1007/s10072-025-08454-7.

## Introduction

Klippel-Feil syndrome (KFS) is a rare congenital malformation characterized by the fusion of at least two cervical vertebrae, first described by Maurice Klippel and André Feil in 1912 [1]. This condition features a symptom triad: short neck, low hairline, and limited neck mobility, found in approximately half of all patients [2].

KFS prevalence has been estimated to be 1 to 40,000, with a slight female predominance, but recent studies suggest a higher occurrence. KFS is the result of the mutation of genes involved in early embryogenesis. KFS is due to a failure of the regular segmentation of the cervical spine vertebrae in fetal development upon the first week of pregnancy [2]. Although many KFS cases are sporadic, hereditary forms with recessive or dominant transmission have been described. Different chromosomes and, consequently, different genes have been implied. GDF6, GDF3, and MEOX1 mutations are the most reported [3, 4] (see Online Resource Table 1). MYO18B has been recently discovered and associated with a specific KFS phenotype, i.e., characteristic facies, myopathy, short stature, and microcephaly [5].

KFS is divided into three types according to the original classification, and it is based on the extension and the location of the vertebral fusions and their association with other spine abnormalities [6]. Type I is associated with the fusion of many cervical and upper thoracic vertebrae. In contrast, type II shows occipital-atlantal fusion and merging of two or three vertebrae or other cervical abnormalities. Finally, type III exhibits cervical fusion associated with lower thoracic or lumbar vertebral fusion [5, 6]. Clarke et al. carried out an updated classification in 1998 [7].

In KFS, neurological deficits are relatively common, primarily due to spinal stenosis, cervical spinal deformity, and vertebral instability. Radiculopathy due to nerve root irritations and chronic compression of the cervical spinal cord with myelopathy signs complete the clinical presentation. Tetraplegia has also been reported. Mirror movements (i.e., simultaneous involuntary contralateral hand movements) have also been described. Cranial nerve abnormalities could be present due to the stretch of their course or anomalies of the pons and medulla [8].

In the report, we illustrate a case of Klippel Feil syndrome with peculiar abnormalities and a disconnect between the extent of the anatomical and neuroimaging alteration and a relatively mild clinical presentation. The case highlights the importance of compensatory neuronal plasticity, even in severe congenital syndrome.

## Case presentation

**Fig. 1:**
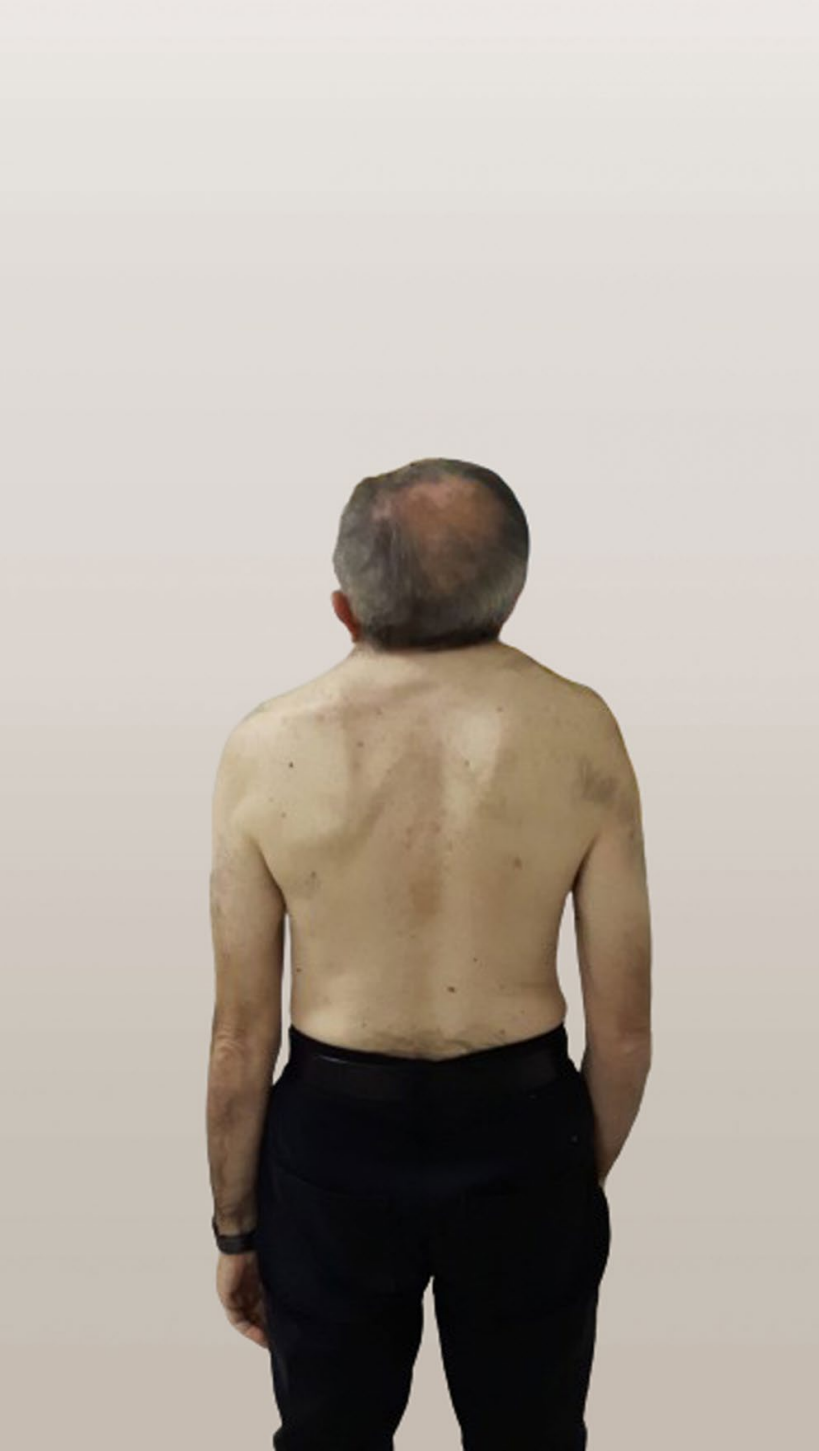
Klippel-feil syndrome triad. A typical KFS triad with a short neck, low hairline, and limited neck range of motion was evident during the patient’s inspection

A 66-year-old right-handed man was referred to our neurological outpatient clinic. The man worked as a professional nurse and was an accomplished amateur drummer. He was independent and married with healthy offspring (two males). The patient complained of gait instability, dizziness after laughing, and progressive motor impairment. He also exhibited mild hearing loss. The neurological examination revealed multidirectional gaze-evoked nystagmus, more prominent on the left side and inextinguishable (see Online Resource Video 1). No cranial nerve impairment was found. Examination showed normal strength and sensory functions. Hyperreflexia in the lower limbs was present and associated with the Babinski reflex. Ataxic gait was not evident, but the subject could not walk in tandem. At the inspection, a typical physical KFS appearance was found (see Fig. 1), and a KFS diagnosis was suspected.

**Fig. 2:**
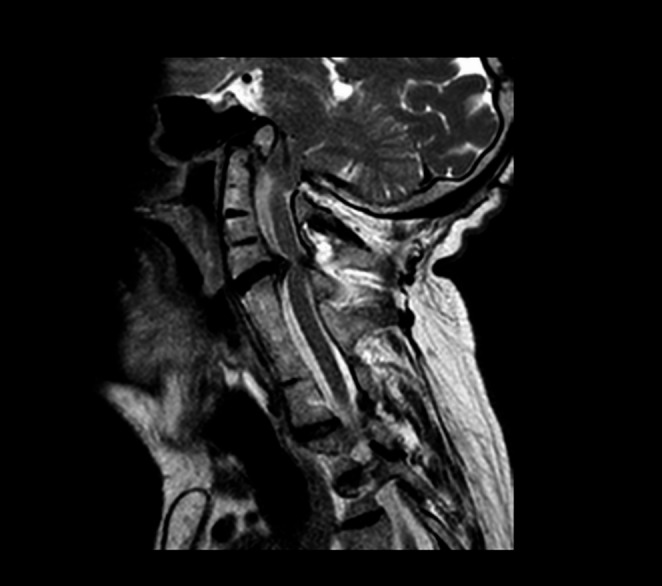
MRI scan of cervical spine. T2-weighted, sagittal view MRI of the cervical spine showed a complex cervical-occipital malformation. The fusion of multiple cervical vertebrae, associated with spinal canal stenosis and C4-C5 disc degeneration, was also evident

**Fig. 3:**
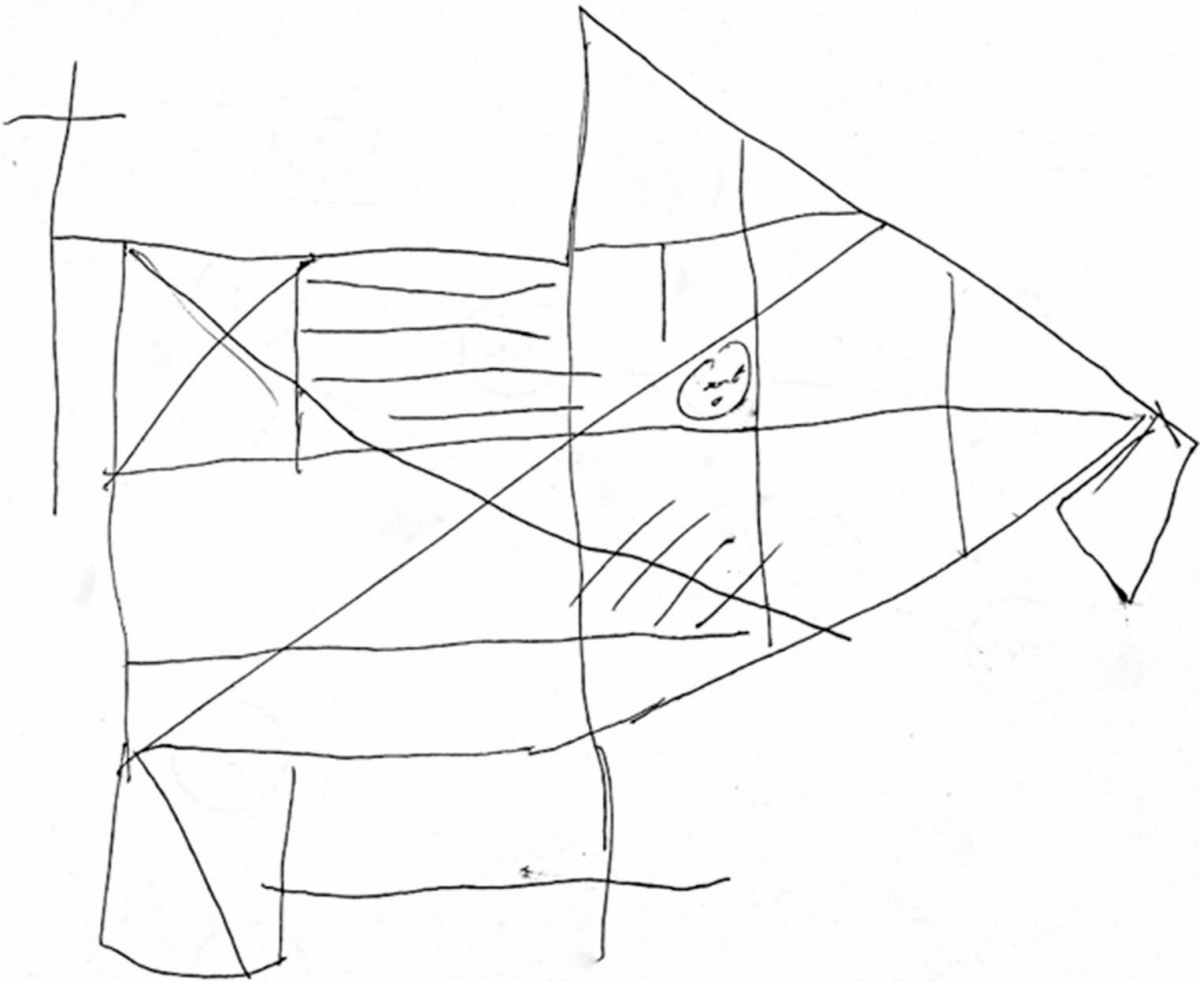
Rey-Osterrieth complex figure test (copy). ROCF copy performed by the patient during the one-year follow-up neuropsychological battery

The patient underwent an MRI scan of the brain and the spinal cord (Fig. 2). The exam showed a complex cervical-occipital malformation, including the rise of the epistropheus above the line of Chamberlain, the fusion of the posterior arch of the atlas with the occipital squama, basilar impression, and downwards dislocation of the cerebellar tonsils. Platibasia (or basilar impression), in which the occiput appeared to be pushed superiorly by the cervical spine, was defined by calculating the skull base angle that measured more than 143°. A brainstem backward dislocation and compression were also found. The medulla and the pons appeared compressed on the right lateral wall by a meningioma, independently from KFS syndrome. A little lipoma was also described behind the colliculus. The most significant alterations appeared in the spine. There was the fusion of the posterior arch of the atlas with occipital squama and the fusions of posterior-lateral elements of multiple cervical and upper thoracic vertebrae (from C2 to C5 and from C6 to D3). Critical spinal canal stenosis deriving from C4-C5 disc degeneration without myelopathy was also present. A radicular cyst was reported. Finally, an ectopic kidney was found. According to the neuroradiological study, KFS type I was diagnosed Fig. 3.

The patient also underwent neuropsychological evaluation (see Online Resource Table 2), in which all performances were normal except for lower-range visuospatial abilities observed at the Rey-Osterrieth complex figure test (ROCF) copy and recall (Image 3) and cognitive flexibility at the Trail Making Test (TMT). The impairment at the ROCF was confirmed at the one-year follow-up visit, whereas the performance at TMT was improved. Thus, while performance on the ROCF appears to reflect a genuine cognitive deficit, the baseline TMT scores were likely influenced by a combination of factors such as general psychophysical condition, transient confounders, and natural intra-individual variability. This highlights the importance of not relying solely on baseline assessment but consistently including follow-up evaluations to distinguish true cognitive deficits from pseudo-deficits. On this occasion, he also underwent Ekman’s Face Recognition test, which showed an overall normal performance in every emotion sub-score except for fear, which was borderline (see Table 1).

**Table 1:** Neuropsychological evaluation (one-year follow-up). The table shows the cognitive performances of the patient at neuropsychological evaluation performed one year later. The Rey complex figure test copy showed persistent deficit results, confirming slight impairment in visual-spatial ability. Equivalent score: 0 = Impairment; 1 = Borderline; 2 = Below average; 3 = Average; 4 = Above average

Domain	Test	Raw score	Adjusted score	Equivalent score	Normal value
Global cognition	Montreal Cognitive Assessment	29	-	-	> 26
Memory	Rey Auditory Verbal Learning Test - Immediate - Delayed	42 9	46 10.3	4 4	> 28.53 > 4.69
	Rey-Osterrieth Complex Figure Test - recall	11	12.5	2	> 9.47
Visuospatial ability	Clock drawing test	10	-	-	≥6
Executive functions	Frontal Assessment Battery (FAB)	18	18	4	> 13.5
Praxis	Rey-Osterrieth Complex Figure Test - copy	25	26.5	0	> 28.88
Language	Letter Fluency	38	39.1	4	> 17.35
	Semantic Fluency	39	43	4	> 25
Attention	Stroop Test - Time interference - Error interference	34.5” 3	24.5” 1.5	4 2	< 36.91 < 4.23
	Trail Making Test (A-B)	A: 48” B: 78” B-A: 30”	31” 10” 0”	4 4 4	< 93 < 282 < 187
Attention in daily activities	Activities of daily living	6/6	-	-	-
	Instrumental activities of daily living	5/5	-	-	-
Ekman test	Global score - Surprise - Happiness - Fear - Disgust - Anger - Sadness	50/60 10/10 10/10 3/10 8/10 9/10 10/10	53.67 - - - - - -	4 - - - - - -	< 49.96 < 6 < 9 < 2 < 4 < 5 < 4

## Discussion

The patient was diagnosed with KFS syndrome type I, according to the original classification, due to the critical fusions of the cervical spine and the involvement of the upper thoracic vertebrae. Multiple congenital anomalies are associated with this syndrome, such as Sprengler’s anomaly, scoliosis, spina bifida occulta, deafness, kidney anomalies, and congenital heart disease, in a significant percentage of people affected [3]. Our patient presented mild hearing impairment, scoliosis, and an ectopic kidney but can live everyday life without limitations and perform complex motor skills despite the complex anatomical malformations. Neuroplasticity, i.e., the capacity of the nervous system to remodel and reorganize itself in response to damage [9], can explain this incredible discrepancy. Notably, neuroplasticity’s effect can be observed after brain and spinal cord injuries, and beyond age barriers, it can manifest in congenital, perinatal, and early development conditions [9, 10]. Therefore, it is possible to observe its remarkable effect on development disorders and other acquired conditions like cerebral palsy or spinal cord injury among infants and young children [10].

Although no association between KFS and visuospatial impairment has been reported, the alteration could be explained by the involvement of GDF6, one of the genes related to KFS [11]. Specifically, GDF6 has been described as controlling the formation of a visual topographic map called retinotectal mapping [11]. This map links through different pathways the retinal output cells and optic tectum midbrain neurons organizing information related to visual stimuli in a spatially ordered manner. Moreover, it preserves the spatial arrangement of retinal ganglion cells through these pathways [12]. Therefore, the retinotectal map is crucial to processing and interpreting visual information by maintaining the spatial relationship between different features of the visual scene [12].

More recently, mutations in three members of the transforming growth factor-β (TGF-β) superfamily (BMP4; GDF6, also known as BMP13; and GDF3) have been associated with microphthalmia or anophthalmia [11, 13]. Interestingly, a specific variant within the myosin-18B (MYO18B) emerged as inversely related to mathematical ability, and the individuals carrying this risk allele exhibited thinner areas of the right parietal cortex, particularly in the right intraparietal sulci [14, 15].

In the present report, the neuropsychological profile of our patient was investigated on the premise that spinal anomalies are embryo-genetically driven by underlying neural tube defects, which are also linked to the developing central nervous system. Accordingly, the presence of atlanto-occiptal anomalies in KFS suggests the possibility of early neurodevelopment abnormalities affecting the immediately adjacent and phylogenetically associated structures. For this reason, considering the anatomical continuity between the occipital bone and the adjacent visual cortex, the detection of lower-range visuospatial functions, compared to the other cognitive functions, is not completely unexpected. Therefore, regardless of the specific underlying genetics, it would be advisable to routinely conduct a comprehensive neuropsychological evaluation on every KFS patient to systematically investigate visuospatial disorders. Early detection could be important because, although it would not cause considerable implications in daily life, it could expose patients to difficulties in complex activities such as driving vehicles.

## Conclusions

Despite the extent of the underlying malformation, subjects with KFS may have a normal life and even perform complex motor tasks, as in the case of our patient, who can play drums at semiprofessional levels. Our patient’s borderline visuospatial functions could be related to the genetic background linking skeletal and visual development deficits, and the impairment in this field, even slight, suggests additional cognitive endophenotypes of this complex syndrome. Finally, our case demonstrates remarkable levels of compensatory neuroplasticity that can also occur in the presence of major congenital defects. In summary, the case exemplifies the complex interplay between genetic factors, anatomical development, and adaptive neural mechanisms in congenital syndromes like KFS.

## Supplementary Information

Below is the link to the electronic supplementary material.

Supplementary Material 1 (DOCX 201 KB)
