# Supplementary material for: Neurological and neuropsychological correlates of Klippel-Feil syndrome
Source: Neurol Sci. 2025 Sep 18;46(11):6047–51. doi: 10.1007/s10072-025-08454-7 (PMC12537603; doi:10.1007/s10072-025-08454-7)
Supplement: Supplementary file 1 — Supplementary Material 1 (DOCX 201 KB) [file 10072_2025_8454_MOESM1_ESM.docx]

**Supplementary material**

**Table 1. Main genetic mutations associated with KFS**

| **Gene** | **Molecular Function** | **Role in physiology** | **Pathological conditions associated** |
| --- | --- | --- | --- |
| **GDF6**  Growth Differentiation Factor 6 | Encodes for a ligand of TGFβ receptors | embryogenesis of bones and joints of the skull, spinal cord, and limbs.  development of the retinotectal map. | Autosomal dominant KFS, coloboma, microphthalmia  Leber congenital amaurosis (LCA 17) |
| **GDF3**  Growth Differentiation Factor 3 | Encodes for a ligand of TGFβ receptors | Skeletal and ocular development. | Autosomal dominant KFS, cervical and thoracic scoliosis, Rudimentary 12th ribs, unilateral iris, microphtalmia and retino-choroidal coloboma. |
| **MEOX1**  Mesenchyme Homeobox 1 | Encodes for homeobox protein involved in embryonic development | differentiation of mesenchymal cells into various tissues, including bones and cartilage. | Autosomal recessive KFS |
| **MYO18B**  Myosin-XVIIIb | protein-coding gene. It belongs to the myosin superfamily of molecular motor proteins. | Cytoskeletal motor activity, cell motility, muscle contraction, and cytokinesis.  Depth reduction of intraparietal sulcus implicated in mathematic-related task. | Autosomal recessive KFS with myopathy and facial dysmorphism  Mathematical disabilities (dyscalculia). |

**Table 1.** The table shows the primary genetic mutation associated with KFS and their physiologic role. The GDF6 gene is implicated in various skeletal and ocular abnormalities and also in major visual defects such as Leber congenital amaurosis: LCA type 17.

**Table 2. Neuropsychological evaluation (baseline)**

| **Domain** | **Test** | **Raw score** | **Adjusted score** | **Equivalent score** | **Normal value** |
| --- | --- | --- | --- | --- | --- |
| Global cognition | Montreal Cognitive Assessment | 26 | - | - | >26 |
| Memory | Rey Auditory Verbal Learning Test   - Immediate - Delayed | 34  7 | 35.3  7.6 | 2  3 | >28.53  >4.69 |
|  | Rey-Osterrieth Complex Figure Test - recall | 10.5 | 14 | 3 | >9.47 |
| Executive functions | Frontal Assessment Battery (FAB) | 18 | 18 | 4 | >13.5 |
| Praxis | Rey-Osterrieth Complex Figure Test - copy | 29 | 29.75 | 1 | >28.88 |
| Language | Letter Fluency | 37 | 33.9 | 4 | >17.35 |
|  | Semantic Fluency | 46 | 46 | 4 | >25 |
| Attention | Stroop Test   - Time interference - Error interference | 16.5”  0 | 11”  0 | 4  4 | <36.91  <4.23 |
|  | Trail Making Test (A-B) | A: 70”  B: 240”  B-A: 170” | 57”  203”  146” | 2  1  1 | <93  <282  <187 |
| Attention in daily activities | Activities of daily living | 6/6 | - | - | - |
|  | Instrumental activities of daily living | 5/5 | - | - | - |

**Table 2.** The table shows the cognitive performances of the patient at neuropsychological evaluation. The patient showed normal cognitive functions in almost all domains except for the Trail Making Test and Rey Complex Figure Test Copy, underlying slight difficulties in visual-spatial ability. Equivalent score: 0= Impairment; 1= Borderline; 2= Below average; 3= Average; 4= Above average.

**Video 1. Neurological examination**

**
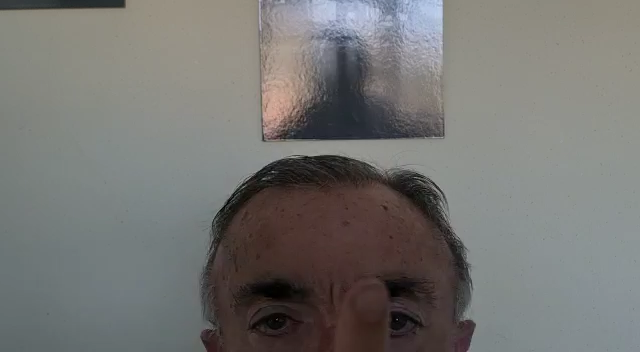
**

**Video 1.** Inexhaustible gaze-evoked nystagmus was revealed at the neurological examination.
